# Enforced Expression of Hoxa3 Inhibits Classical and Promotes Alternative Activation of Macrophages In Vitro and In Vivo

**DOI:** 10.4049/jimmunol.1501944

**Published:** 2016-06-24

**Authors:** Hadeel Al Sadoun, Matthew Burgess, Kathryn E. Hentges, Kimberly A. Mace

**Affiliations:** Faculty of Life Sciences, University of Manchester, Manchester M13 9PT, United Kingdom

## Abstract

The regulated differentiation of macrophages (mφs) and their subsequent activation into proinflammatory or prohealing subtypes is critical for efficient wound healing. Chronic wounds such as diabetic (db) ulcers are associated with dysregulation of macrophage function. Whereas non-db mφs polarize to an M2-like, prohealing phenotype during the late stages of healing, db-derived mφs continue to display an M1-like, proinflammatory, or a mixed M1-like/M2-like phenotype. We have previously shown that sustained expression of *Hoxa3* reduces the excessive number of leukocytes within the db wound; however, the effect of Hoxa3 on mφ polarization was unknown. In this study, we show that Hoxa3 protein transduction of mφs in vitro enhances macrophage maturation, inhibits M1 polarization, and promotes M2 polarization, in part via regulation of Pu.1/Spi1 and Stat6. Sustained expression of *Hoxa3* in vivo in db wounds reduces the number of Nos2^+^ (M1-like) mφs, increases the number of Arg1^+^ and VEGF^+^ (M2-like) mφs, and accelerates healing in a DNA-binding independent manner. Our findings suggest a role for Hox protein activity in promoting M1-to-M2-like phenotypic switching via interactions with myeloid transcription factors and provide insight into mechanisms regulating this process in db wound healing.

## Introduction

The number of patients suffering from chronic wounds is nearing epidemic proportions. Patients with both type 1 and type 2 diabetes have impaired wound healing, and 15% of these patients go on to develop chronic, nonhealing wounds, 84% of which result in lower limb amputation (reviewed in Ref. 1). Current treatments are not effective because of a poor understanding of the mechanisms underlying chronic wounds; thus, they are the leading cause of nontraumatic amputations in the developed world today (2, 3). During the past two decades, the role of dysregulated inflammation in impaired healing associated with aging and diabetes has become apparent. This is due in part to increased and prolonged expression of proinflammatory cytokines and chemokines, such as TNF and IL-1 (4) as well as a decrease in growth factors such as fibroblast growth factors, TGF-β, platelet-derived growth factor, and vascular endothelial growth factor (VEGF), which normally function to promote new tissue growth and may also act as anti-inflammatory factors (5–8). However, therapies targeted at manipulating these factors alone are not sufficient to control chronic inflammation, and only a mild improvement in wound healing has been observed in the clinic. Therefore, a stronger focus on reprogramming cell behavior in the wound, in particular the therapeutic manipulation of macrophages (mφs), could be an effective strategy.

mφs are involved in all phases of wound repair and regeneration (9, 10). During the early stages of healing, proinflammatory (M1-like) mφs dominate the wound environment, where they are involved in killing pathogens and removing debris. As the wound progresses to late stages of healing, most mφs acquire the prohealing (M2-like) state that is critical for healing progression (11–13). In mice, the M2-like state is characterized by high levels of arginase production (11, 14). M2 mφs can be generated in vitro by the stimulation of the IL-4R and/or IL-13R, which signal via the phosphorylation of Stat6 (15–17). This pathway is also important to wound healing in vivo (18). However, the transition of proinflammatory mφs to prohealing mφs is inhibited by pathological environments, such as aging (19) or diabetes. In addition, diabetes affects the process of how progenitor cells mature in the bone marrow (BM), how myeloid cells infiltrate the wound tissue, and how mφs respond to signals from the environment (13, 20, 21). Mφs from diabetic (db) mice continue to display these defects even when cultured outside the db environment, showing an aberrant polarization response to both pro- and anti-inflammatory stimuli (12, 13, 22–24). Extrinsic signals coming from local cells and Th2 lymphocytes, including IL-4, TGF-β, and IL-10 are insufficient to induce the phenotypic switch from M1-like to M2-like mφs (13, 22). This deficit in reparative mφs is associated with decreased angiogenesis, extracellular matrix remodeling and wound contraction (reviewed in Ref. 25). Therefore, there is a need to identify factors that can stimulate the phenotypic switch of mφs within the wound from a proinflammatory to a prohealing phenotype to promote resolution of inflammation and wound healing.

Enforced expression of *Hoxa3* has been shown to promote resolution of inflammation and wound healing in db wounds (21, 26). Hoxa3 is a member of the homeobox gene family which encode master regulator transcription factors that specify segmental identity along the anterio-posterior axis (27). In addition, many Hox genes are expressed in early hematopoietic progenitors where they direct lineage commitment, cell proliferation, and self-renewal (28–30). Hox proteins can be delivered to cells directly via their capacity to cross-biological membranes in a receptor- and energy-independent manner (31, 32). Importantly, protein transduction of Hox proteins is a potentially safer method than gene transfer. For example, Hoxb4 protein transduction can promote hemopoietic stem cell self-renewal without inducing leukemia (33).

Enforced Hoxa3 expression in hematopoietic progenitors was shown to promote their differentiation into Gr1^+^CD11b^+^ cells, a proangiogenic myeloid population, and rescue their diabetes-induced defects (34). However, the specific effects of enforced Hoxa3 on mφs were unknown.

We found that Hoxa3 protein transduction had an effect on both mφ maturation and activation and attenuated the inflammatory phenotype of mφs, possibly via regulation of Spi-1/Pu.1, an Ets family transcription factor that is required for the generation of mature mφs (35–38). Our data show that Hoxa3 inhibits M1-like/proinflammatory gene expression and induces expression of markers of M2-like/prohealing mφs as well increasing Stat6 phosphorylation, showing a link between Hoxa3 function and the IL-4 signaling pathway. Furthermore, sustained expression of *Hoxa3* in vivo in db wounds also promotes the switch from an M1-like, proinflammatory mφ phenotype to an M2-like, prohealing mφ phenotype. These findings have therapeutic relevance because this switch in mφ phenotype is a key event in efficient wound healing (13, 24).

## Materials and Methods

### Animals

All animals were housed at the University of Manchester animal care facility, and all procedures were approved by the local ethical review committee and the Home Office and were performed in accord with the requirements of the Animal (Scientific Procedures) Act 1986. *Lepr^db/db^*, the most well-characterized mouse model of type 2 diabetes, and heterozygous control *Lepr^db/+^* mice were purchased from Harlan (Oxfordshire, U.K.) or The Jackson Laboratory (Bar Harbor, ME). All animals used were 8–16 wk old and were age- and sex-matched to controls. A minimum of three biological replicates per group were used for in vitro experiments. For in vivo wound healing studies, six mice per group were used.

### Wound model

*Lepr^db/db^* (type 2 diabetes model) and heterozygous control *Lepr^db/+^* (non-db) mice were anesthetized, and the dorsum was shaved and sterilized with antiseptic wipes. Eight millimeter in diameter full thickness wounds were excised including the *panniculus carnosus* layer. Plasmid DNA (either empty vector or containing wild-type or mutant *Hoxa3*) was mixed 1:1 with 1% methyl cellulose in water, and 25 μg in a volume of 50 μl was spotted and air-dried to form a pellet. Animals received buprenorphine at the time of surgery, and pellets were applied to open wounds. Animals were housed in separate cages until tissue was harvested at the described time points by removing the entire wound area, including a 2-mm perimeter, or for wound healing studies, wounds were measured at the indicated time points using planimetry. Wound areas were calculated using Adobe Photoshop, and statistics were performed using Microsoft Excel.

### Culturing of BM-derived mφs

BM from tibia and femurs of type 2 db and control non-db mice was flushed into DMEM (Sigma-Aldrich), passed through a 19-gauge needle and a 70-μm filter (BD Falcon) to generate a single-cell suspension. A total of 1 × 10^6^ cells/ml were plated in mφ medium (DMEM supplemented with 10% FBS [Sigma-Aldrich]), 1× penicillin/streptomycin (Sigma-Aldrich), 10% L929 cell (American Type Culture Collection)–conditioned medium containing M-CSF on day 0. Cells were allowed to differentiate for 7 d at 37°C under 5% CO_2_. Fresh medium was added on day 3; the medium was changed completely on day 5.

### Overexpression of Hoxa3 in mouse mφs via protein transduction

The HEK293T cell line (American Type Culture Collection) was maintained in DMEM supplemented with 10% FBS and 1× penicillin/streptomycin. At 20% confluency, cells were transfected with 10 μg SP-Hoxa3-mCherry (SP-Hoxa3^mCh^) or SP-mCherry expression plasmids using the calcium phosphate method (39), and live imaging was used to monitor transfection efficiency. Conditioned medium (CM) from the transfected cells was collected at 24, 48, and 72 h posttransfection, filtered via a 0.45-μm filter, and used to supplement mouse mφs in culture at a 1:1 volume with mφ medium on days 5 and 6 of differentiation. Hoxa3^mCh^ in the medium entered mφs via passive translocation, as previously described in Refs. 31–34.

### Detection of Hoxa3-mCherry protein in CM and mouse mφs

Lysates from mφs treated with Hoxa3^mCh^ or mCherry CM were prepared using IP lysis buffer (50 mM Tris-acetate [pH 7.5], 300 mM NaCl, 1 mg/ml BSA, 2% Igepal, 1 mM EDTA, 1× protease inhibitor mixture [Promega], and 1× sodium orthovanadate). Two micrograms of rabbit anti-mCherry (BioVision) was labeled with biotin (DSB-X; Molecular Probes) and incubated with 50 μg protein lysate, together with 0.4 mg prewashed Dynabeads (FlowComp Flexi system; Invitrogen). The mixture was left overnight at 4°C on a rotator before washing and elution with immunoprecipitation wash buffer (0.1% SDS, 500 mM Tris-acetate [pH 7.5], 300 mM NaCl, and 0.5% Igepal) using a magnet (EasySep; StemCell Technologies).

CM from 293T cells transfected with *SP-Hoxa3^mCh^* or *SP-mCherry* and purified lysate from mφs treated with CM were analyzed by Western blotting using 10% SDS-PAGE and polyvinylidene difluoride membrane, using the Bio-Rad Turbo Transfer System. Blots were probed at 4°C overnight with 0.1 μg rabbit anti-mCherry Ab in a volume of 6 ml and visualized using the Pierce ECL detection system (Thermo Fisher Scientific). Images were generated using the ChemiDoc MP System (Bio-Rad) and Image Lab Software.

### Flow cytometry analysis

BM-derived mφs (BMDMs) were trypsinized and resuspended in flow cytometry buffer (PBS with 2% FBS). Following this, 1 × 10^6^ cells were blocked with 0.5 μg rat anti-mouse CD16/32 (2.4G2; BD Pharmingen) in a volume of 100 μl for 30 min on ice. Cells were then stained with the following Abs: 5 μg/ml eFluor450 anti-F4/80 (BM8; eBioscience), 1.25 μg/ml PE Cy7 anti-CD115 (25-1152; eBioscience), or 1.2 μg/ml eFluor450 anti-CD11b (M1/70; eBioscience). Flow cytometry was performed with a Fortessa LSR II (BD Biosciences) and analyzed using FlowJo software.

### RNA extraction and cDNA synthesis

RNA was isolated from wound- mφs and/or BMDMs at the specified time points by cell or tissue homogenization in TRIzol (Life Technologies). RNA was treated using the DNase I enzyme at room termperature for 10 min (Qiagen). One microgram of RNA was used for the reverse transcription reaction using Bioscript RT (Bioline) and random primers in a total volume of 25 μl. The resulting cDNA was diluted to 1:15 to be used as a template for quantitative RT-PCR (qRT-PCR).

### Gene expression analysis

qRT-PCR analysis was performed using the following TaqMan Gene Expression Assays: *Itgam* (Mm00434455_m1), *Emr1* (Mm00802529_m1), *Csf1r* (Mm01266652_m1), *Ym1*/*Chi313* (Mm00675889_m1), *Arg1* (Mm00475988_m1), *Tgfβ* (Mm01178820_m1), *Mrc1* (Mm01329362_m1), *Nos2* (Mm01309897_m1), *Tnf* (Mm00443258-m1), *Cd86* (Mm00444543-m1), *Ccl2* (Mm00441242_m1), *Spi1* (Mm00488142_m1), *Stat6* (Mm01160477_m1), and *Socs1* (Mm00782550-s1). *Hsp90ab1* (Mm00833431_g1) and *Hist2h2aa1* (Mm00501974_s1) were used as reference genes. Reactions were performed on a StepOnePlus cycler (Applied Biosystems) and analyzed using the δ-δ Ct method. All analyses were performed using Microsoft Excel 2013.

### Coimmunoprecipitation of Hoxa3 and Spi-1/Pu.1 protein

293T cells cotransfected with 5 μg pSport6-Pu.1 and pcDNA3.1-Hoxa3mCherry were harvested 48 h posttransfection. Cell lysates obtained using 1% Triton X-100 and 0.01% Igepal in PBS. Two micrograms of rabbit anti-mCherry (BioVision) and/or goat anti-Pu.1 (D-19; Santa Cruz Biotechnology) were labeled with biotin (DSB-X). Cell lysates (300 μg) were incubated with biotinylated anti-mCherry or anti-Pu.1 and then mixed with prewashed Dynabeads, according to the manufacturer’s instructions. The eluate was then analyzed by Western blot.

### Luciferase reporter assay

RAW 264.7 mφs (ATCC-LGC Standards) were treated with 20% M-CSF and 50% CM from either mCherry or Hoxa3^mCh^ cultures for 24 h. Cells were transfected with 1 μg pMAX enhanced GFP (eGFP), 1 μg pSport6-Pu.1, and 1 μg pGL2-7.2*fms* luciferase vector (40) with a 1:4 ratio of FuGENE HD transfection reagent (Promega) and incubated for 24 h. Cell eGFP expression was measured by fluorescence microscopy, and luciferase activity assayed using the Luciferase Assay System (E1500), according to the manufacturer’s protocol (Promega). The level of luciferase activity is shown as relative light units calculated as light units/mean eGFP fluorescence.

### mφ activation

Hoxa3^mCh^- or mCherry-treated mφs from db or non-db mice were split into the following three groups: 1, nonactivated mφs; 2, mφs that were classically activated (CA) using serum-free mφ medium supplemented with 100 ng/ml LPS (Sigma-Aldrich) and 100 ng/ml IFN-γ (Sigma-Aldrich); and 3, mφs alternatively activated (AA) using serum-free medium supplemented with 20 ng/ml IL-4 (PeproTech) and 50 μg/ml anti–IFN-γ. Twenty-four hours postactivation, RNA was harvested from each group independently for gene expression. Forty-eight hours postactivation, cells were harvested or supernatants collected for arginase assays, ELISA, and Greiss assays.

### NO release (Greiss assay)

NO release was determined using supernatants from mφs with Greiss reagents, as described previously (41). A colorimetric plate reader was used to measure absorbance at 570 nm and background absorbance at 630 nm.

### Arginase assay

Arginase activity in mφ lysates was measured using the spectrophotometric method, as described previously (42). Briefly, 10 mM MnCl_2_ and 0.5 M l-arginine were sequentially added to the mφs and incubated for 1 h at 37°C. The reaction was stopped by the addition of an acid solution, and the formation of urea by arginase was analyzed by adding isonitrosopropiophenone at 100°C for 45 min. The colored product was measured at 570 nm and the background absorbance at 450 nm.

### ELISA

Detection of mouse TNF-α and IL-12 was determined using a cytokine-specific ELISA kit (eBioscience) as described previously (13). For mouse TGF-β, a Ready-Set-Go mouse TGF-β ELISA kit (eBioscience) was used according to the manufacturer’s instructions.

### Wound processing for immunofluorescence staining

Wounds were processed from three type 2 db or control non-db mice treated with CMV-Hoxa3 or plasmid control as described in (19). Wounds were stained as described previously, with some modifications (13). In brief, 5 μm sections were generated from formalin-fixed wounds and stained with H&E using a Shandon Varistain 24-4 (Thermo Scientific) to locate the granulation tissue and peri-wound dermis. Matching sections were labeled with the following Abs diluted in PBS + 0.1% Tween 20 (PBT): rat anti-Mac3 (1:100, clone M3/84 (RUO); BD Pharmingen), goat anti-Arg1 (1:200, sc-18354; Santa Cruz Biotechnology), rabbit anti-Nos2 (1:100, sc- 651; Santa Cruz Biotechnology), rabbit anti-Vegf (1:400, EP1176Y; Abcam), and rabbit anti-Tgfβ (1:150, EPR12678(B); Abcam). Next, the following secondary Abs were applied: donkey anti-rat Alexa Fluor 488 (1:500, A-21208; Invitrogen Life Technologies), donkey anti-goat Cy5 (1:500, ab6566; Abcam), and donkey anti-rabbit Alexa Fluor 555 (1:500, A-31572; Invitrogen Life Technologies). Four fields from each wound were imaged for statistical analysis of Mac3^+^Nos2^+^Arg1^−^, Mac3^+^Nos2^+^Arg1^+^, or Mac3^+^Nos2^−^Arg1^+^. For VEGF and TGF-β staining, the number of Mac3^+^, VEGF^+^, and Mac3^+^VEGF^+^ or Mac3^+^, TGFβ^+^, and Mac^+^TGFβ^+^ cells were determined. All sample analyses were performed using Imaris 3D/4D image processing and analysis software (Bitplane). The percentage of each phenotype was calculated as the mean ± SEM of all images in each category (*n* = 12).

### Western blotting

Whole-cell lysates were obtained from mouse mφs at the specified time points by lysing the cells in complete radioimmunoprecipitation lysis buffer (1% Triton X-100, 1% sodium deoxycholate, 0.1% SDS, 1 mM PMSF, 2 mM sodium orthovanadate, and 1× protease inhibitor mixture [Fisher, BPE9706-1] in PBS). Phosphatase inhibitor was added to the lysis buffer when harvesting cell lysates for pStat6 detection. Cell lysate (10 μg) was subjected to 10% SDS-PAGE and transferred to polyvinylidene difluoride membrane. The blot was incubated with either goat polyclonal anti-Pu.1 (1:200, D-19, sc-5949; Santa Cruz Biotechnology), rabbit monoclonal anti-Stat6 (1:2000, YE361, ab32520; Abcam), or rabbit polyclonal anti-pStat6 (1:1000, Tyr^641^, ab195647; Abcam). Protein was detected using ECL (Thermo Fisher Scientific).

## Results

### Protein transduction of Hoxa3 in mouse mφs promotes maturation

**FIGURE 1.**
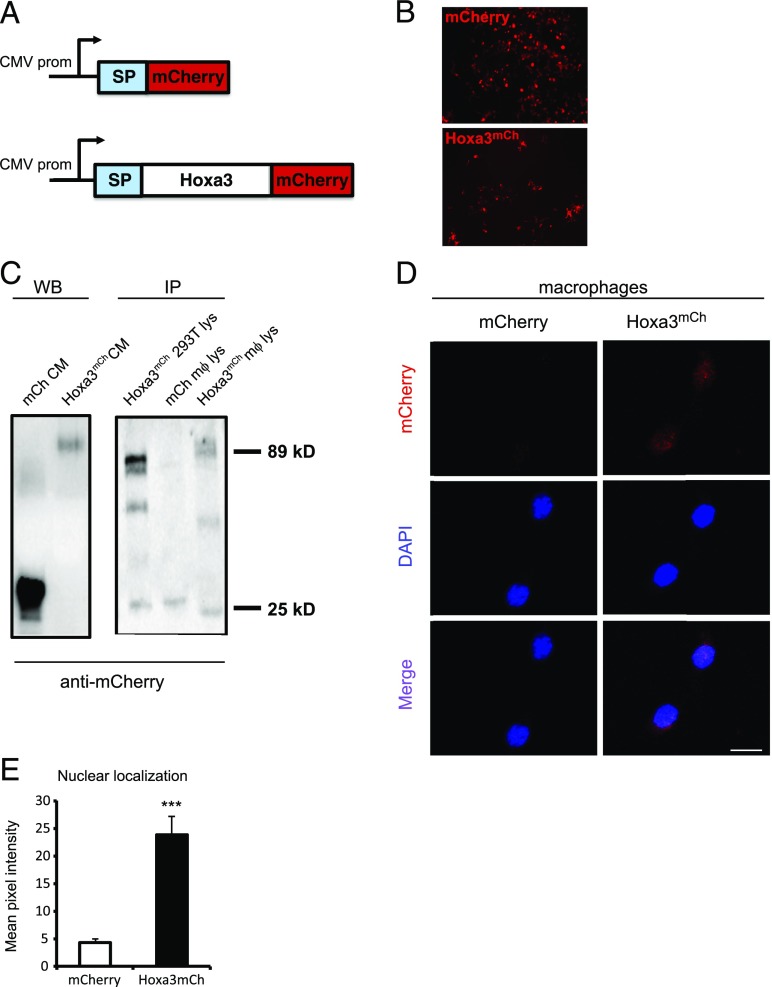
Protein transduction of Hoxa3^mCh^ in mouse mφs. (**A**) Schematic representation of signal peptide (SP)-mCherry control and SP-Hoxa3^mCh^ constructs. Expression is directed by the *CMV* promoter (prom). (**B**) Fluorescent images of mCherry and Hoxa3^mCh^ protein expression in transfected 293T cells. (**C**) Western blot detection of Hoxa3^mCh^ and mCherry (mCh) in CM (left blot) and anti-mCherry immunoprecipitated lysate (lys) from 293T cells transfected with Hoxa3^mCh^ (right blot, left lane), mouse mφs treated with CM containing mCherry (right blot, middle lane), or Hoxa3^mCh^ (right blot, right lane), respectively. (**D**) Representative confocal image of mφs treated with mCherry CM (left panels) or Hoxa3^mCh^ CM (right panels) showing anti-mCherry immunolocalization (top panels), DAPI staining (middle panels), and merged channels *(bottom panels*). (**E**) Quantification of nuclear localization of mCherry and Hoxa3^mCh^ following treatment with the respective CM, *n* = 40 mφs. Original magnification ×400. ***p < 0.001.

We and others have shown that mφs isolated from the db environment show differentiation and maturation defects, which likely contribute to their dysfunction in vivo (13, 22). Sustained expression of *Hoxa3* in wounds of type 2 db mice results in significant reduction in the total number of inflammatory cells within the wound (21), but the mechanism underlying this effect on mφs was not identified. As protein transduction of Hoxa3 in hematopoietic stem cell/progenitors was shown to promote their differentiation into Gr1^+^CD11b^+^ cells, a proangiogenic granulocytic–monocytic cell population (34), we aimed to test whether overexpression of Hoxa3 by protein transduction on monocyte/mφ precursors from type 2 db mice could rescue their differentiation defects. To test this, we used a construct in which Hoxa3 is fused to mCherry and to the Ig κ-chain signal peptide (Fig. 1A) to allow the secretion of Hoxa3 into the medium (43). Transfection efficiency of Hoxa3-mCherry (Hoxa3^mCh^) and mCherry control expression plasmids in 293T cells was monitored by live imaging (Fig. 1B). The presence of Hoxa3^mCh^ and mCherry protein in the CM and in the targeted mouse mφs was confirmed via immunoprecipitation (Fig. 1C) and confocal imaging (Fig. 1D), respectively. Quantification of Hoxa3^mCh^ and mCherry control protein localization within mφs was performed to validate appropriate nuclear localization of the Hoxa3^mCh^ protein (Fig. 1E).

**FIGURE 2.**
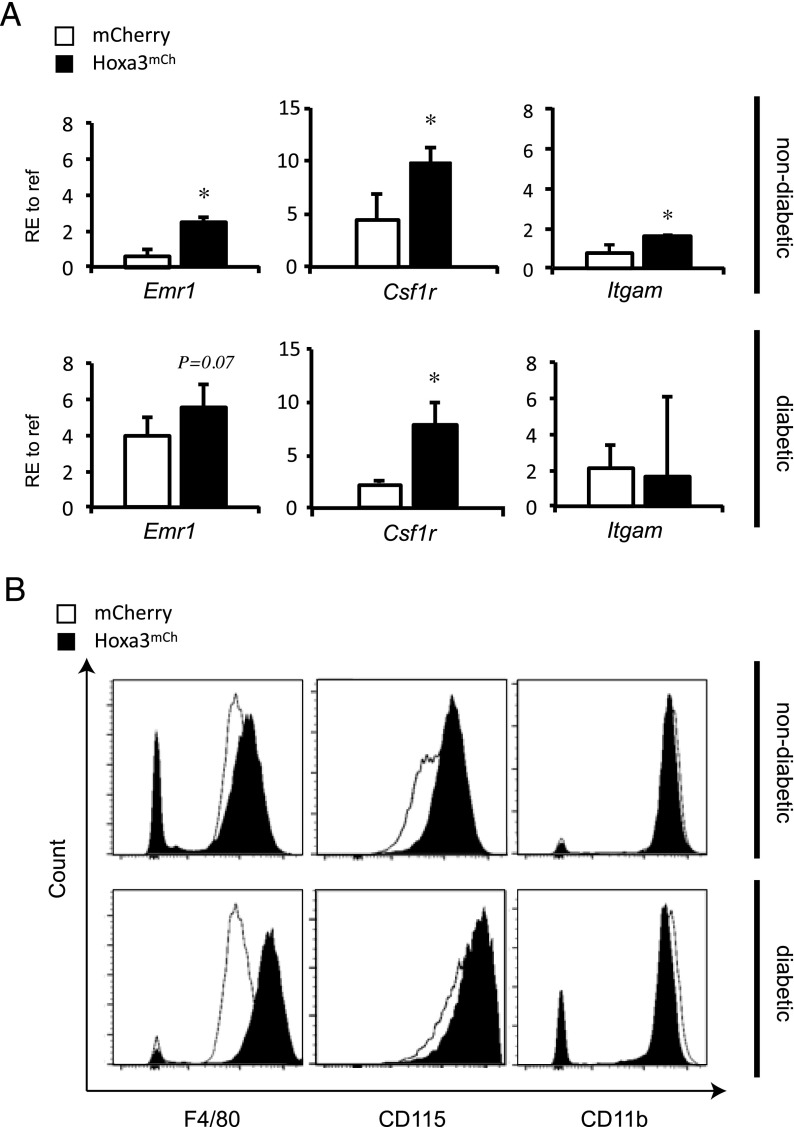
Analysis of mφ maturation following Hoxa3^mCh^ protein transduction. (**A**) qRT-PCR analysis of mφ maturation markers *Emr1* (encoding F4.80), *Csf1r* (encoding CD115/M-CSFR), and *Itgam* (encoding CD11b) in non–db- and db-derived mφs treated with either Hoxa3^mCh^ (▪) or mCherry control (□) CM for 24 h (RE, relative expression to reference gene, *n* = 3, error bars = SEM; *p < 0.05). (**B**) Representative histogram plots from flow cytometry analysis of same markers in (A) in non–db- (top panels) and db-derived (bottom panels) mφs treated with either Hoxa3^mCh^ (black shading) or mCherry control (white) CM for 24 h.

Following Hoxa3^mCh^ or mCherry control protein transduction for 24 h, total RNA was isolated from target mφs and qRT-PCR conducted to measure expression of *Emr1*, encoding F4/80 (a mφ-specific marker), *Itgam*, encoding CD11b (common myeloid marker), and *Csf1r*, encoding the M-CSF receptor (CD115) to assess mφ maturation. Mφs derived from non-db and db mice showed significant increases in *Emr* (*p* < 0.05 and *p* = 0.07, respectively; Fig. 2A) and *Csf1r* (*p* = 0.05) in response to Hoxa3^mCh^. A significant increase in *Itgam* (*p* < 0.05) in the non–db-derived population was observed, but there was no apparent change in the db-derived mφs (Fig. 2A). We also analyzed changes in expression of these markers at the protein level by flow cytometry (Fig. 2B). Protein transduction of Hoxa3^mCh^ showed a trend of increased mean fluorescence intensity (MFI) of cell surface F4/80 protein in non–db-derived mφs (Hoxa3^mCh^ MFI = 27725.5 ± 13624 versus mCherry MFI = 17380.8 ± 13690, *p* = 0.2) and in the db-derived population (Hoxa3^mCh^ MFI = 11746 ± 7681 versus mCherry MFI 8913 ± 5499, *p* = 0.1). CD115 was significantly increased by Hoxa3^mCh^ protein transduction in non–db-derived mφs (Hoxa3^mCh^ MFI 10188.5 ± 2577 versus mCherry MFI 6213 ± 962, *p* = 0.05), and db-derived mφs (Hoxa3^mCh^ MFI = 35915 ± 11729 versus mCherry MFI = 21824 ± 5971, *p* < 0.1). However, no detectable changes were noted in CD11b levels between Hoxa3^mCh^-treated and mCherry-treated mφs. Altogether, the Hoxa3-mediated regulation of mφ maturation markers suggests that Hoxa3^mCh^ protein transduction promotes the maturation of non–db- and db-derived mφs. The discrepancies in transcript and protein levels of *Itgam* may be due to posttranscriptional regulation of this protein, which has been shown in the db environment (13).

### Hoxa3 upregulates Spi1 levels in mouse mφs

**FIGURE 3.**
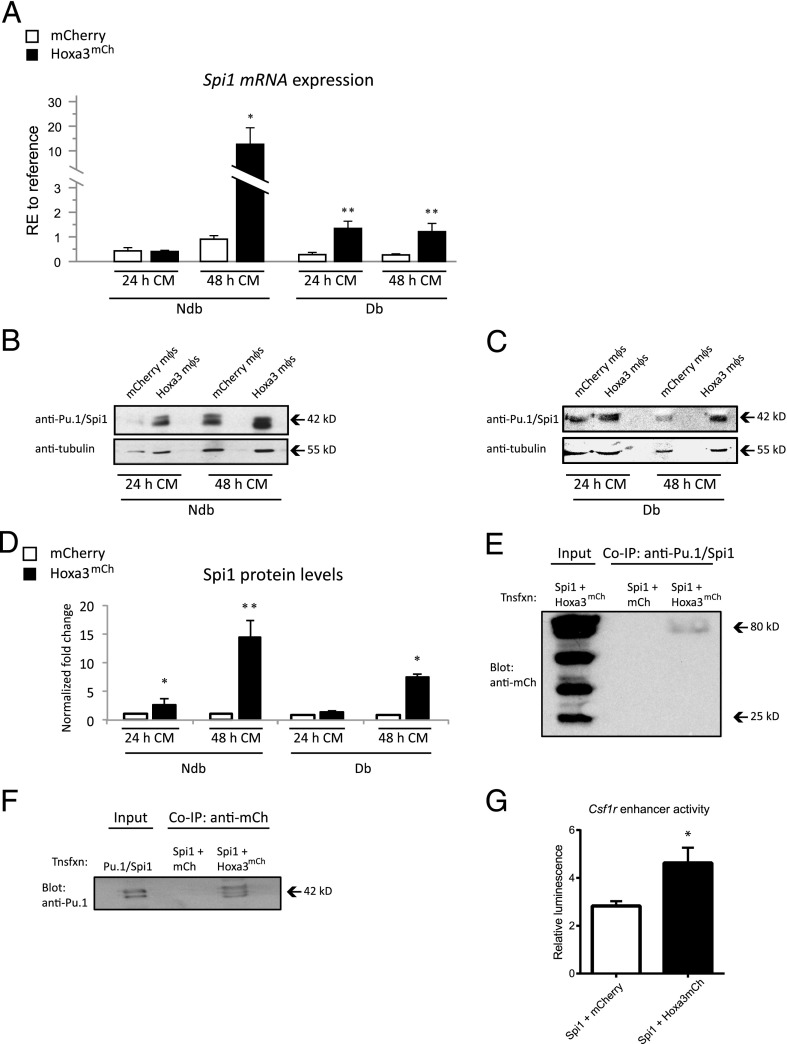
Hoxa3 protein transduction increases *Spi1* mRNA and protein levels in mouse mφs and physically interacts with Spi1 protein. (**A**) *Spi1* expression after 24 or 48 h of control (mCherry, □) or Hoxa3^mCh^ (▪) protein transduction in non–db (ndb)- or db-derived mφs (RE, relative expression to reference gene, *n* = 3, error bars = SEM; *p < 0.05, **p < 0.01). Representative Western blot analysis of Spi1 levels in control-treated (mCherry) or Hoxa3^mCh^-treated ndb (**B**) or db (**C**) mφs. (**D**) Quantification of Spi1 protein levels from Western blots as shown in (B) and (C). Spi1 levels were normalized to tubulin levels on same blot (*n* = 3, error bars = SEM; *p < 0.05, **p < 0.01). (**E**) Coimmunoprecipitation (Co-IP) of Spi1 and Hoxa3^mCh^ proteins in transfected 293T cells. Left lane shows whole-cell lysate input from 293T cells transfected with *Spi1* and *Hoxa3^mCh^* expression plasmids, lane 2 shows Co-IP using anti-Pu.1/Spi1 Ab from cells cotransfected with negative control (*mCherry*) and Spi1 expression plasmids, and lane 3 shows Co-IP using anti-Pu.1/Spi1 Ab from cells cotransfected with *Hoxa3^mCh^* and *Spi1* expression plasmids. Blot was probed with anti-mCherry Ab. The predicted size of Hoxa3^mCh^ is 73 kDa and mCherry is 26 kDa. (**F**) Co-IP of Spi1 and Hoxa3^mCh^ proteins in transfected 293T cells. Left lane shows whole-cell lysate input from 293T cells transfected with *Spi1* and *Hoxa3^mCh^* expression plasmids, lane 2 shows Co-IP using anti-mCherry Ab from cells transfected with *Spi1* and control plasmids, and *lane* 3 shows Co-IP using anti-mCherry Ab from cells transfected with *Spi1* and *Hoxa3^mCh^* expression plasmid. Blot was probed with anti-Pu.1/Spi1 Ab. The predicted size of Spi1/Pu.1 is 31 kDa. (**G**) Hoxa3 promotes Spi1/Pu.1 activation of the *Csfr1* enhancer. Luciferase report activity in RAW 264.7 macrophages cotransfected with pGL2-7.2*fms* luciferase reporter plus *Spi1* expression plasmid and treated with mCherry or Hoxa3^mCh^ protein CM. Luciferase activity was normalized to fluorescence from eGFP transfection control, and values represent mean ± SEM of *n* = 4 experimental replicates. *p < 0.05. mCh, mCherry; Tnsfxn, transfection.

Spi1 (also known as Pu.1), Cepbα, and Runx1 transcription factors are critical for monocyte and mφ differentiation and are known to upregulate expression of myeloid differentiation markers, including *Csf1r* and *Itgam* (44). We hypothesized that Hoxa3 may promote maturation of murine mφs via modulating the function of Spi1, Cepbα, and/or Runx1 transcription factors. We first assessed whether any differences were evident in the levels of expression of these transcription factors between non-db and type 2 db mφs. The db-derived mφs showed a significant reduction in *Spi1* (*p* < 0.05; Supplemental Fig. 1A), whereas *Cebpa* and *Runx1* levels did not show significant differences among the two populations (Supplemental Fig. 1B, 1C). We then assayed whether Hoxa3^mCh^ protein transduction for 24 or 48 h could affect *Spi1*, *Cebpα*, or *Runx1* mRNA levels. Interestingly, Hoxa3 upregulated *Spi1* mRNA levels in non–db-derived mφs after 48 h (*p* < 0.05; Fig. 3A) and in the db-derived mφs at 24 h (*p* < 0.05) and 48 h (*p* < 0.005; Fig. 3A) but did not affect the levels of *Cebpα or Runx1* (data not shown). We further investigated whether the changes observed in *Spi1* mRNA levels were consistent with the Spi1/Pu.1 protein levels in non–db- and db-derived mφs. Surprisingly, Spi1/Pu.1 protein levels in non-db mφs were significantly increased at 24 h following Hoxa3^mCh^ treatment, despite a lack of significant increase in mRNA at this time point. However, as expected at 48 h following Hoxa3^mCh^ treatment, we detected a ∼14.8-fold increase in Spi1/Pu.1 protein (Fig. 3B). In the db-derived mφs treated with Hoxa3^mCh^, Spi1/Pu.1 protein levels were also significantly increased by ∼1.5-fold at 24 h and by ∼7.4-fold at 48 h compared with control-treated cells (Fig. 3C). Levels of Spi1/Pu.1 were quantified relative to tubulin (Fig. 3D, 3E). These data suggest that Hoxa3 may induce Spi1/Pu.1 at the transcriptional and posttranscriptional levels. This Hoxa3-mediated upregulation of Spi1/Pu.1 protein in db-derived mφs may be one mechanism by which Hoxa3 promotes mφ maturation.

### Hoxa3 and Pu.1 proteins physically interact

We next investigated whether Hoxa3 may physically interact with the Pu.1/Spi1 transcription factor and function as a cofactor on myeloid differentiation target genes. Other Hox proteins, such as Hoxc13, have been shown to interact with ETS proteins, including Pu.1/Spi1, by physical and functional interactions that are implicated in the differentiation of erythroid leukemia cell lines (27). We examined biochemical interactions between Pu.1 and Hoxa3 by performing immunoprecipitation assays on cell lysates from 293T cells that were cotransfected with mCherry-tagged Hoxa3 and Pu.1. The anti-Pu.1 Ab coimmunoprecipitated Hoxa3 but not the mCherry-only control (Fig. 3E), whereas the anti-mCherry Ab coimmunoprecipitated Pu.1/Spi1 in the Hoxa3^mCh^-Spi1 cotransfection only (Fig. 3F), suggesting that Hoxa3 and Pu.1/Spi1 proteins physically interact.

To test whether this interaction affects Spi1 DNA binding or regulation of its target genes, we analyzed Spi1 activation of two *Csf1r* enhancers using a luciferase reporter assay system. Spi1 has been shown to physically bind to two enhancer regions near the *Csfr1* promoter, one upstream of the transcriptional start site and one downstream, in the first intron, and abolition of this binding results in loss of enhancer activity (45, 46). We found that protein transduction of Hoxa3 in combination with Spi1 significantly promoted *Csfr1* enhancer activity when both sites were present (pGL2-7.2*fms* construct) in response to M-CSF compared with control protein transduction (Fig. 3G). However, protein transduction of Hoxa3 did not affect the upstream-only reporter activation (pGL-0.*5fms* construct), suggesting it promotes Spi1 activation through the downstream enhancer (data not shown).

### Hoxa3 inhibits M1 proinflammatory polarization of BMDMs

**FIGURE 4.**
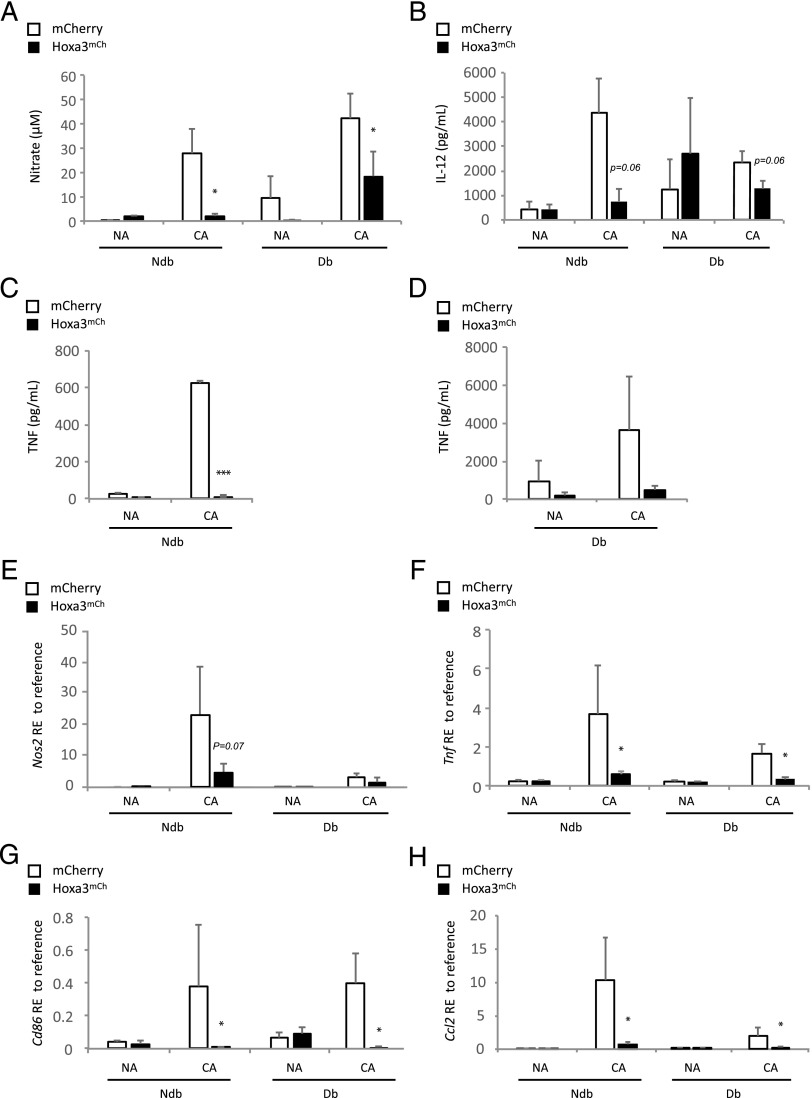
Analysis of proinflammatory (M1) mφ markers in Hoxa3^mCh^- or mCherry-treated BMDMs from non-db (ndb) and db mice. (**A**) Production of NO (as measured by nitrate as the final product in the Griess assay) in NA or CA mφs from ndb or db mice and treated with mCherry (□) or with Hoxa3^mCh^ (▪). (**B**) IL-12 release from NA or CA mφs from ndb or db mice, treated with mCherry (□) or Hoxa3^mCh^ (▪). (**C** and **D**) TNF release from NA or CA mφs from ndb and db mice, respectively, treated with mCherry (□) or with Hoxa3^mCh^ (▪). (**E**–**H**) Relative expression (RE) of *Nos2*, *Tnf*, *Cd86*, and *Ccl2* to *Hsp90* (reference gene, reference, *n* = 5; *p < 0.05, ***p < 0.005).

One of the reported functions of Hoxa3 in accelerated healing of the type 2 db mouse model is reducing the total number of inflammatory cells recruited to or retained in the wound via downregulation of NF-ĸB pathway genes (24). The NF-ĸB pathway promotes proinflammatory gene expression and an M1-like phenotype. On the basis of these results, we wished to test the ability of Hoxa3 to control the proinflammatory phenotype of mφs. Non–db- and type 2 db–derived mφs treated with Hoxa3^mCh^ or mCherry CM were either left in a naive state (nonactivated [NA]) or CA using LPS and IFN-γ. Production of NO as well as the proinflammatory cytokines TNF and IL-12 were then assessed. We found that Hoxa3^mCh^ significantly reduced NO production from CA mφs (Fig. 4A). In addition, the level of IL-12 cytokine production was reduced in CA Hoxa3^mCh^-treated non–db-derived mφs by more than 4-fold compared with control-treated cells (Fig. 4B). In db-derived mφs, Hoxa3 also inhibited IL-12 production in CA mφs by ∼2-fold compared with control-treated cells (Fig. 4B). In a similar manner, Hoxa3 markedly reduced TNF production by ∼53-fold in CA non–db-derived mφs (Fig. 4C) and showed a trend in reducing TNF production in CA db–derived mφs compared with controls (*p* = 0.18; Fig. 4D).

To determine whether protein transduction of Hoxa3 can limit the expression of M1 marker genes in CA mφs, we assessed the expression of M1 markers in NA and CA db and non–db-derived mφs (Fig. 4E–H). Hoxa3^mCh^-treated CA mφs showed significantly reduced expression of all M1 marker genes tested including NO synthase (*Nos2*; Fig. 4E), *Tnf* (Fig. 4F), chemokine C-C motif ligand 2 (*Ccl2*) (Fig. 4G), and *Cd86* (Fig. 4H). These data, therefore, support the hypothesis that enforced expression of Hoxa3 can limit M1 mφ polarization in the presence of proinflammatory stimuli such as LPS and IFN-γ.

### Hoxa3 promotes M2 prohealing polarization of BMDMs

**FIGURE 5.**
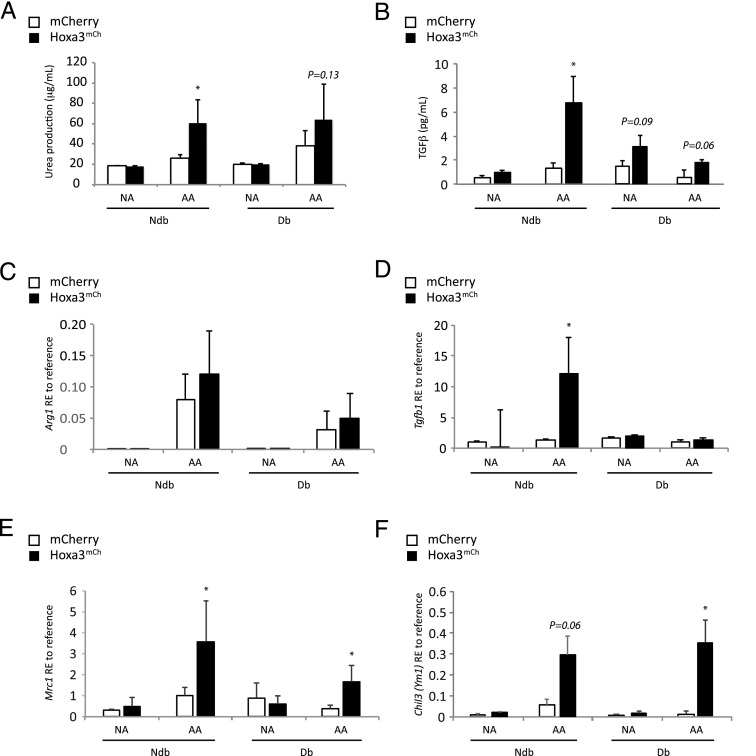
Analysis of prohealing (M2) mφ markers in Hoxa3^mCh^- or mCherry-treated BMDMs from non-db (ndb) and db mice. (**A**) Arginase assay (urea production from l-arginine) in NA or AA mφs from ndb or db mice treated with mCherry (□) or with Hoxa3^mCh^ (▪). (**B**) TGF-β production from NA or AA mφs from ndb or db mice treated with mCherry (□) or Hoxa3^mCh^ (▪). (**C**–**F**) Relative expression (RE) of *Arg1*, *Tgfb1*, *Mrc1*, and *Chi3l3(Ym1)* to *Hsp90* (reference gene, *n* = 5; *p < 0.05, ***p < 0.005).

Prohealing mφs (M2-like) are the major source of growth factors such as TGF-β that contribute to tissue repair and wound healing (8). Previous work from our laboratory has shown that enforced expression of Hoxa3 upregulates *Tgfb* in myeloid cells (34), suggesting it may promote M2-like polarization. Therefore, we assessed the profile of Hoxa3^mCh^-treated mφs for M2 markers such as arginase and TGF-β production. Arginase, a hallmark of AA mφs in mice, was assessed by measuring urea in cell lysates, which is a product from arginase degradation of l-arginine. This assay showed significantly higher levels of urea in AA non–db-derived mφs and a trend of increased urea levels in AA db–derived mφs treated with Hoxa3^mCh^ compared with control-treated mφs (Fig. 5A). Hoxa3 also increased the levels of anti-inflammatory cytokine/growth factor TGF-β by ∼5-fold in AA non–db-derived mφs (Fig. 5B) and by ∼2.9-fold in AA db–derived mφs (*p* = 0.06; Fig. 5B). Further analyses of the regulatory effect of Hoxa3 protein transduction on levels of these M2 markers were obtained by performing qRT-PCR on *Arg1*, *Mrc1* (mannose receptor CD206), *Tgfb*, and chitinase 3-like 3 (*Chi3l3*, also known as *Ym1*) on RNA extracted from NA and AA mφs treated with Hoxa3^mCh^ or mCherry control CM. Hoxa3^mCh^-treated AA non–db- and db-derived mφs showed significant upregulation of *Tgfb*, *Mrc1*, and *Chi3l3* mRNA in AA non–db- and db-derived mφs (Fig. 5C–F). Altogether, the data suggest that Hoxa3 promotes M2 polarization by reducing M1 markers and upregulating M2 markers, which may contribute to its capacity to promote wound resolution in vivo.

### Hoxa3 inhibits M1-like and promotes M2-like polarization of macrophages in day 7 db wounds

**FIGURE 6.**
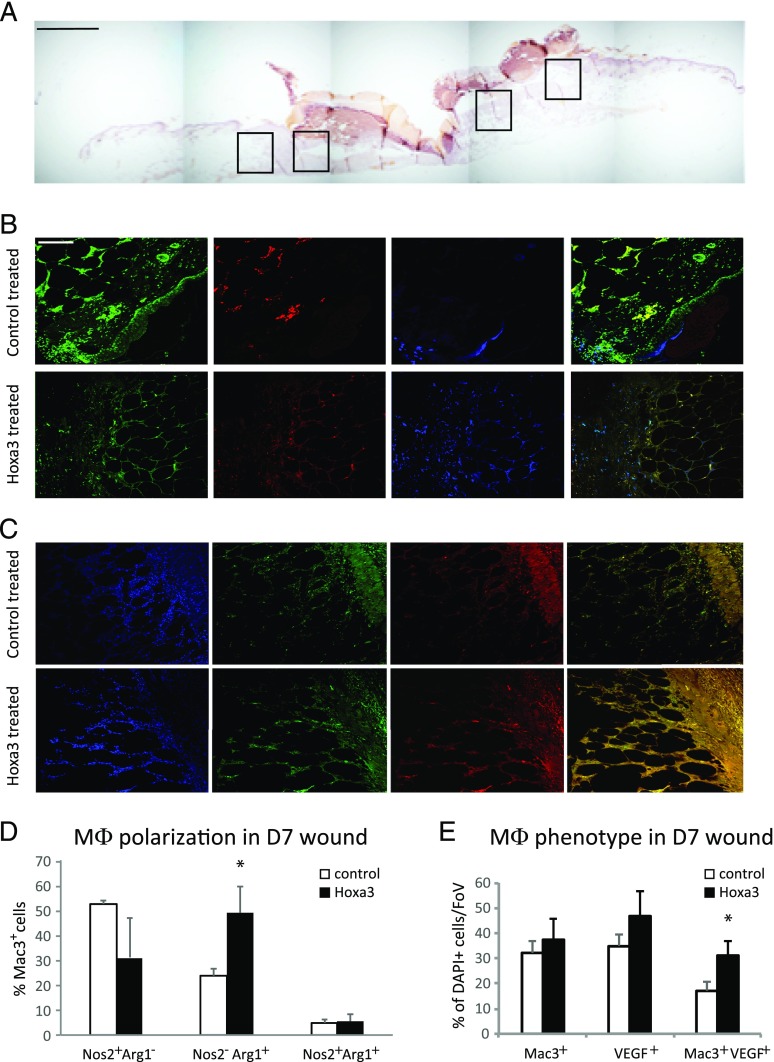
Enforced expression of Hoxa3 in vivo inhibits M1-like polarization and promotes M2-like polarization in wounds of db mice. (**A**) Representative section from day 7 wound of db mouse showing areas of analysis (indicated by boxes; scale bar, 1 mm). (**B** and **C**) Representative images of immunofluorescent (IF) detection of Mac3 (mφ marker; green), Nos2 (proinflammatory marker; red), and Arg1 (prohealing marker; blue) in (B) and DAPI (blue), Mac3 (green), and VEGF (prohealing mφ marker; red) in (C) from day 7 wound of db mouse treated with empty vector control or with *Hoxa3* expression plasmid (scale bar, 10 μm). (**D**) Quantification of Mac3^+^Nos2^+^, Mac3^+^Arg1^+^ and Mac3^+^Nos2^+^Arg1^+^ cells in IF sections from (B). (**E**) Quantification of Mac3^+^, VEGF^+^, and Mac^+^VEGF^+^ cells in IF sections from (C); *p < 0.05. FoV, field of view.

The data above suggested enforced expression of Hoxa3 suppress the M1 phenotype and promote the M2 phenotype in cultured mφs. We therefore wished to investigate whether treating type 2 db wounds with Hoxa3 could influence mφ phenotype in vivo. Previous studies from our laboratory found that wounds of db mice show significant increases in M1-like (Nos2^+^) cells and significant decreases in M2-like (Arg1^+^) cells when compared with non-db wounds at day 7 following wounding (13). Because day 7 in the healing process represents the phase where most mφs in non-db wounds have transitioned from a proinflammatory phenotype to a prohealing phenotype, we therefore investigated the effect of treating db mouse wounds with *Hoxa3* at this time point. Sections were taken from full thickness excisional wounds that were treated with *Hoxa3* or control and subsequently labeled with Abs for detecting Mac3 (pan-macrophage marker), Nos2 (M1 marker), and Arg1 (M2 marker). Analysis was performed on granulation tissue and peri-wound dermis (Fig. 6A). In *Hoxa3*-treated wounds, 7 d postinjury, triple staining for Mac3, Nos2, and Arg1 showed a trend of reduced Nos2^+^Arg1^−^ (M1-like) mφs in the Hoxa3^mCh^-treated wound as compared with control treated and significantly increased numbers of Nos2^−^Arg1^+^ (M2-like) mφs (Fig. 6B, 6D). There appeared to be no difference in the number of mixed phenotype Nos2^+^Arg1^+^ mφs.

Additional markers for proreparative mφs, TGF-β and VEGF, were also analyzed, as several previous studies have shown that TGFβ^+^ and VEGF^+^ macrophages are critical for effective healing (12, 47). Sections from db wounds treated with *Hoxa3* or control were double labeled with Abs detecting Mac3 (mφ marker) and VEGF or TGF-β, and analysis was performed as above. In *Hoxa3*-treated wounds, 7 d postinjury, double staining for Mac3 and VEGF showed a significant increase in the number of Mac3^+^VEGF^+^ mφ populations in diabetic wounds (*p* < 0.05; Fig. 6C, 6E). However, although *Hoxa3* significantly increased the number of total TGFβ^+^ cells in the wound (*p* < 0.05), it did not increase the Mac3^+^TGFβ^+^ subpopulation (Supplemental Fig. 2A, 2B), suggesting that these populations may be partially distinct. Negative controls for all Abs are shown in supplementary material (Supplemental Fig. 3A–C). Altogether, the data in this paper provide evidence for Hoxa3 promoting an M2-like phenotype in wound macrophages in vivo.

### Hoxa3 upregulates IL-4 signaling pathway effector Stat6 and increases phospho-Stat6 levels

**FIGURE 7.**
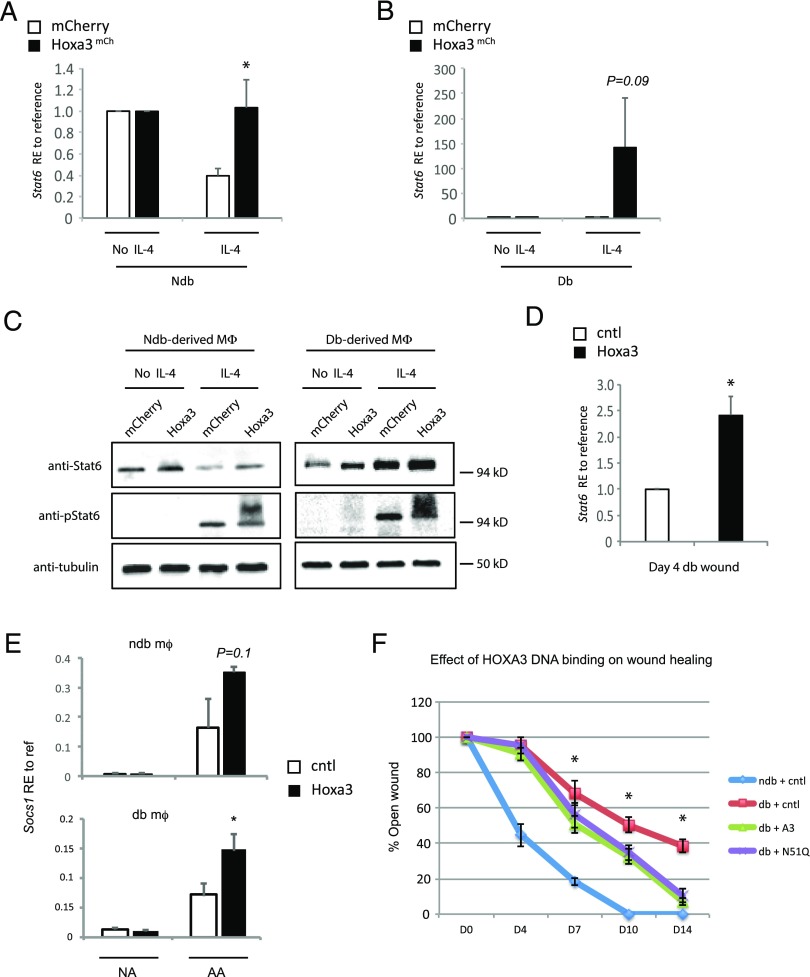
Hoxa3 regulation of IL-4-Stat6/phospho-Stat6 signaling pathway during M2 activation. Relative expression (RE) of *Stat6* in mCherry (□) or Hoxa3^mCh^-treated (▪) non–db (ndb)-derived mφs (**A**) or db-derived mφs (**B**), stimulated with IL-4, normalized to mφs without IL-4 stimulation (no IL-4). *Hist2h2aa1* and *Hsp90ab1* were used as reference genes, and values represent mean ± SEM of *n* = 5 biological replicates. *p < 0.05. (**C**) Representative Western blots of total protein lysates from mφs pretreated with Hoxa3^mCh^ or mCherry CM that were left unstimulated (no IL-4) or stimulated with IL-4 and probed with the indicated Abs. Blots were repeated three times on mφs from three different mice. (**D**) *Stat6* expression in day 4 wounds of db mice treated with empty vector (□) or *Hoxa3* expression plasmid (▪) relative to reference. (**E**) *Socs1* expression in NA and AA ndb-derived mφs (upper graph) and db-derived mφs (lower graph) treated with control (□) or Hoxa3^mCh^ (▪). (**F**) Mean percent open wound following 0.8-cm excisional wounding on days indicated in ndb mice treated with control plasmid (blue line), db mice treated with control plasmid (orange line), db mice treated with wild-type *Hoxa3* plasmid (green line), and db mice treated with mutant *Hoxa3* plasmid encoding a change in homeodomain aa 51 from Asn to Gln (N51Q). Hoxa3, A3, *n* = 6/group, error bars indicate SEM, *p < 0.05 when comparing Hoxa3-treated db versus control-treated db. cntl, control.

The IL-4 signaling pathway strongly promotes M2 macrophage polarization via Stat6 activation. Upon IL-4 stimulation, Stat6 is phosphorylated by the activated JAKs, and translocates through the nucleus to function as a transcription factor that upregulates *Arg1*, *Mrc1*, and *Chi3l3*/*Ym1* (M2 genes) (15, 16). To determine whether Hoxa3 promotes the M2-like mφ phenotype by interacting with the IL-4 signaling pathway, we investigated Stat6 activation following Hoxa3^mCh^ protein transduction in vitro. We assessed the levels of *Stat6* transcript, and total Stat6 protein as well phospho-Stat6 in Hoxa3^mCh^-treated mφs. We found that Hoxa3 upregulated *Stat6* transcript and Stat6 protein levels in both non-db and db-derived mφs following IL-4 stimulation (Fig. 7A–C). Interestingly Hoxa3 increased total Stat6 protein accumulation in the unstimulated cells (Fig 7C), although no detectable transcript difference was apparent (Fig. 7A, 7B), suggesting that Hoxa3 may also mediate stabilization of Stat6 protein. In addition, phospho-Stat6 was also markedly increased in the presence of Hoxa3 in non–db-derived and db-derived mφs (Fig. 7C). Analysis of Hoxa3-treated whole wound samples from type 2 db mice also revealed up-regulation in *Stat6* mRNA levels (Fig. 7D), suggesting *Stat6* is a downstream target of Hoxa3 in vivo as well. We did not observe any change in *Il4* or *Tgfb1* mRNA expression in vivo (data not shown), suggesting that Hoxa3 modulation of Stat6 is independent of these genes. We also analyzed *Socs1* expression in response to Hoxa3, as *Socs1* is known to be activated in response to IL-4 signaling, and is crucial for IL-4–dependent M2 polarization in mφs (48). Protein transduction of Hoxa3^mCh^ significantly increased *Socs1* expression in db-derived mφs and showed a trend of increased expression in non–db-derived mφs (Fig. 7E). Taken together, our findings suggest that enforced expression of Hoxa3 can promote alternative activation of mφs via increasing cellular pools of Stat6 and promoting Stat6 phosphorylation in murine mφs.

### Hoxa3 accelerates wound healing in type 2 db mice in a DNA-binding independent manner

We have previously shown that enforced expression of Hoxa3 in db wounds accelerates wound healing (26). However, our prior work was performed using a larger excisional wound model than the current study. In addition, we wished to test whether DNA binding is required for this function. To do this, we induced a mutation to the reading frame encoding the homeodomain of Hoxa3 to change asparagine 51 to glutamine (N51Q), abolishing DNA binding (49). Interestingly, we found that the N51Q mutant was able to promote wound healing in db mice as effectively as the wild type Hoxa3 (Fig. 7F). This finding is consistent with a model in which Hoxa3 promotes macrophage maturation and polarization via interaction with other DNA-binding partners, such as Spi1/Pu.1.

## Discussion

To assess the therapeutic potential of Hoxa3 on dysregulated mφs, we used an ex vivo culture model of BMDMs from type 2 db and non-db mice. Hox proteins possess the ability to translocate across biological membranes in an energy-free, endocytosis-free manner because of the penetratin peptide located in the third α helix of homeodomain proteins (31, 32). Using this property, we delivered Hoxa3 into cultured mφs using protein transduction. This method is potentially a safe and useful method to overexpress transcription factors ex vivo to reprogram cells for therapeutic use because it does not involve gene manipulation or possible damage to the cell that may be caused by other methods such as viral transduction. The passive translocation of Hox proteins was previously described with Hoxa3 and Hoxb4 using a coculture system to deliver them into hemopoietic stem cells (33, 34). In this study, we demonstrate for the first time, to our knowledge, the successful delivery of Hoxa3 protein into mφs using a CM enriched with Hoxa3 rather than the more complicated coculture system approach. This is superior to a coculture system as it is a step closer to good manufacturing practice conditions, which would be required for potential therapeutic use.

mφs are critical to wound healing as they secrete growth factors and cytokines necessary for wound resident cell proliferation, migration, and other behaviors (12, 50, 51). They also contribute to resolution of the inflammatory phase via efferocytosis (removal of dead/dying cells in the wound), particularly of neutrophils. The presence of neutrophils until late stages of healing in db wounds can negatively impact the healing process by causing oxidative damage and exacerbating tissue damage by secreting or leaking excessive proteases and elastases (52, 53). Khan et al. (54) found that successful efferocytosis can switch the mφ phenotype in the db wound from proinflammatory to anti-inflammatory. Furthermore, several studies have suggested that db-derived mφs fail to perform this function effectively (55–57). However, until now it has not been clear whether delayed/failed maturation of db mφs may underlie defective efferocytosis in db wounds, leading to excessive accumulation of leukocytes. The link between abnormal maturation of myeloid cells and diabetes was demonstrated in non-obese db mice in which administration of GM-CSF promoted the maturation of DCs and subsequently reduced the development of diabetes (58) as well as in obese db mice in which administration of G-CSF–promoted Gr-1^+^CD11b^+^ cell maturation and wound healing (59); thus, the cause and effect relationship between these two states may be intertwined. Our data suggest that this relationship can be extrapolated to macrophages in diabetic chronic wounds as well.

Several studies have demonstrated that diabetes induces a dysfunctional mφ polarization phenotype (13, 22, 60), even when cells are removed from the db environment and cultured ex vivo (13). Although environmental stimuli play a large role in mφ phenotype, this last observation suggests a role for cell intrinsic factors in regulating mφ maturity and phenotype as well. Transcription factors are likely candidates for such cell intrinsic factors because they control cell differentiation and phenotype by regulating hundreds of target genes that affect multiple processes within the cell. Hoxa3 has been shown to be aberrantly regulated in db wounds, and enforced expression of this potent transcription factor can significantly inhibit inflammation, promote angiogenesis, and accelerate healing (21, 26, 34). In this study, we investigated the effect of Hoxa3 protein transduction and in vivo gene transfer on mφ maturation and phenotype both ex vivo and in vivo. Hoxa3-treated mφs showed upregulation of maturation markers F4/80 and CD115 as well a master regulator of mφ development, Spi1/Pu.1, at both transcriptional and protein levels, thus supporting the hypothesis that Hoxa3 rescues diabetes-induced mφ maturation defects. In addition, we found that Hoxa3 can directly interact with Spi1, suggesting Hoxa3 may act as a cofactor enhancing Spi1 function when overexpressed. Upregulation of *Csf1r*, a known downstream target of Spi1, supports this hypothesis. In addition, luciferase assays indicate Hoxa3 collaborates with Spi1 to activate the promoter/enhancer elements controlling *Csf1r* expression. However, transcription of *Itgam*, which encodes CD11b and is another known target of Spi1 (61), was not upregulated by Hoxa3 overexpression, suggesting only a subset of Spi1 targets are modulated by Hoxa3 interactions. Interestingly, abolishment of Hoxa3 DNA-binding did not affect its ability to promote wound healing, providing further evidence that Hoxa3 modulates macrophage behavior via direct interactions with myeloid transcription factors. Further studies to clarify the importance of Hoxa3-Spi1 interactions on mφ maturation and polarization during wound healing will address this question.

In addition to promoting mφ maturation, Hoxa3 attenuated the proinflammatory response of mφs to LPS and IFN-γ and reduced NO, IL-12, and TNF release in cell supernatants. Analysis of M1 gene expression supported the hypothesis that Hoxa3 inhibited M1 polarization. This is consistent with the previously described anti-inflammatory effect of Hoxa3 in vivo, where sustained expression of *Hoxa3* reduced the recruitment and/or retention of inflammatory cells and resulted in downregulation of genes involved in multiple inflammatory pathways, including NF-ĸB (21).

In addition to suppressing the M1 polarization phenotype, enforced expression of Hoxa3 also promotes the M2 polarization phenotype. We found Hoxa3 upregulated *Tgfb*, a marker of M2 mφs, at the mRNA level and increased TGF-β growth factor release from non–db- as well db-derived mφs. This is consistent with a previous study showing upregulation of *Tgfb* in Gr-1^+^CD11b^+^ myeloid cells transfected with *Hoxa3* (34). TGF-β functions during the later stages of wound healing to promote angiogenesis, stimulate myofibroblast differentiation and wound closure, and promote collagen deposition (reviewed in Ref. 62). Hoxa3 treatment of wounds also increased the number of TGF-β–positive cells in the wound, although not specifically in macrophages. Hoxa3 treatment also increased arginase production in mφs from both non-db and type 2 db mice. Arginase is thought to promote wound healing by shifting the production of NO to urea and ornithine. Ornithine is a precursor of polyamines and collagen (reviewed in Ref. 63). Although arginase is not highly expressed in human monocytes or mφs when stimulated in vitro (64), it has been shown to be expressed in human neutrophils (65), which may indicate that these cells play a similar role in human wounds with regard to arginase function. Stat6 function in murine and human mφs is conserved, and thus, our finding that Hoxa3 treatment induces upregulation of *Stat6* and increased phosphorylation of Stat6 will be relevant to human mφ biology. Stat6-dependent gene regulation is critical to M2 mφ behavior (reviewed in Ref. 11).

**FIGURE 8.**
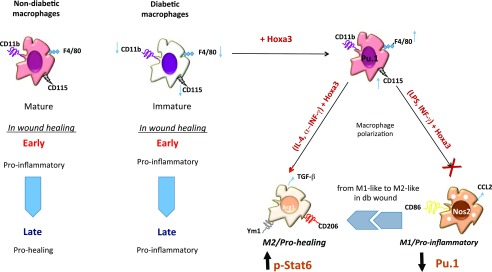
Model of multiple effects of Hoxa3 on db mφs. db-derived mφs have aberrant maturation and dysregulated polarization phenotypes. Enforced expression of Hoxa3 in ex vivo–differentiated mouse mφs promotes differentiation of mφs and increases expression of F4/80 and CD115 via upregulation of the Spi1/Pu.1 transcription factor. Hoxa3 also rescues the aberrant polarization phenotype in db mφs, promoting the M2/prohealing polarization state in BM-derived as well in day 7 db wound mφs. This occurs in part by Hoxa3-mediated increases in cellular pools of Stat6 protein as well as phosphorylation. In parallel, Hoxa3 also inhibits the heightened M1/proinflammatory mφ phenotype in db mφs, possibly by inhibiting NF-κB activation.

Impaired wound healing in both type 1 and type 2 db mouse models and human patients has been linked to a failure in mφs to switch from the M1 phenotype to the M2 phenotype (13, 22, 60). In this study, we have shown that enforced expression of Hoxa3 in type 2 db-derived mφs can promote this switch during wound healing via multiple mechanisms (Fig. 8). Hoxa3 treatment promotes mφ maturation via upregulation of and direct interaction with Spi1, which may lead to enhanced mφ function. This in turn may attenuate proinflammatory responses and augment anti-inflammatory behaviors. Hoxa3 also inhibits M1 polarization via suppression of proinflammatory gene expression and promotes M2 polarization by enhancing IL-4 signaling through increased Stat6 activation. Altogether, these functions of Hoxa3 shift the balance from an M1-dominant wound environment to an M2-dominant wound environment, thus accelerating healing.

We conclude that driving intrinsic changes in mφs are key to overcoming aberrant environmental signals, which are present in tissues displaying chronic inflammation. Using protein transduction may be a potential therapeutic technology to safely reprogram inflammatory cell behavior in vivo. Future studies will be focused on addressing mechanisms underlying Hoxa3-Spi1 interactions and how regulation of mφ maturation may be involved in the M1-to-M2 transition.

## Supplementary Material

Data Supplement
