# Supplementary material for: Enforced Expression of Hoxa3 Inhibits Classical and Promotes Alternative Activation of Macrophages In Vitro and In Vivo
Source: J Immunol. 2016 Jun 24;197(3):872–84. doi: 10.4049/jimmunol.1501944 (PMC4947829; doi:10.4049/jimmunol.1501944)
Supplement: Data Supplement [file JI_1501944.zip › JI_1501944_Supplemental_Figures_1.pdf]

SUPPLEMENTARY FIGURE 1

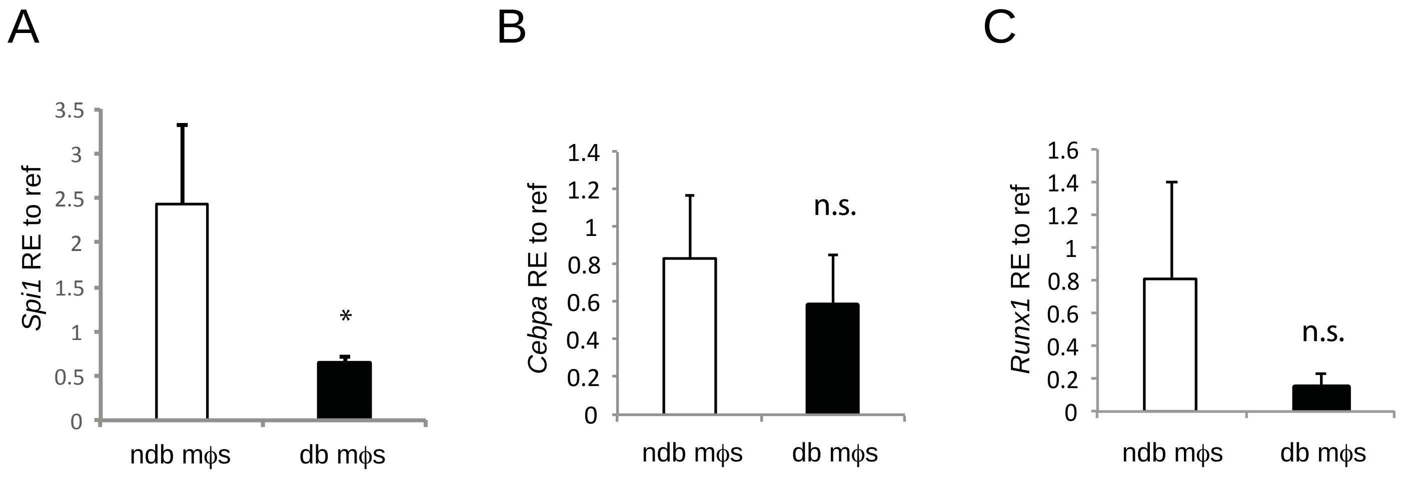

Supplementary Figure 1. Analysis of myeloid transcription factor expression in ndb and db-derived macrophages. Relative expression (RE) of **(A)** *Spi1*, **(B)** *Cebpa* and (C) *Runx1* to reference (ref) gene (*Hist2h2aa1*) in ndb (white bar) and db-derived (black bar) mφs. Non-diabetic, ndb, diabetic, db, macrophages, mφs, n = 3-5 biological replicates per group, **P*<0.05, not significant, n.s.

SUPPLEMENTARY FIGURE 2

A

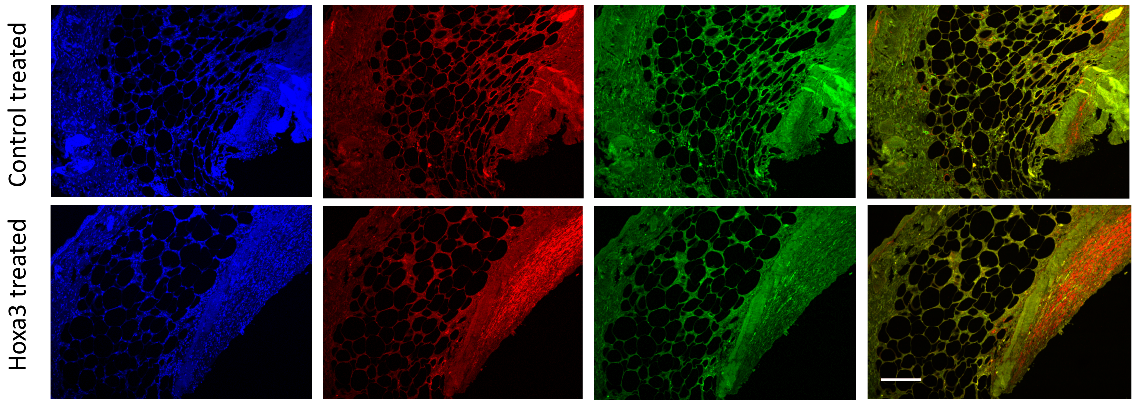

B

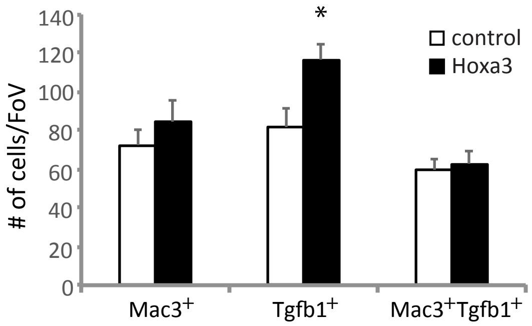

Supplementary Figure 2. Enforced expression of Hoxa3 in vivo increases TGFβ positive cells in wounds of db mice. (A) Representative image of immunoflouresencet detection of Dapi (blue), Mac3 (green), TGF-β (reparative growth factor; red) and Mac3+ TGFβ+ (yellow) in day 7 wound treated with empty vector control or with Hoxa3 expression plasmid. Images taken at 10x magnification (scale bar: 10 μm). (B) Quantification of Mac3+, TGFβ+ and Mac3+ TGFβ+ cells in IF sections from control treated (white) or Hoxa3-treated (black) wounds, n = 6 animals per group, *P<0.05.

SUPPLEMENTARY FIGURE 3

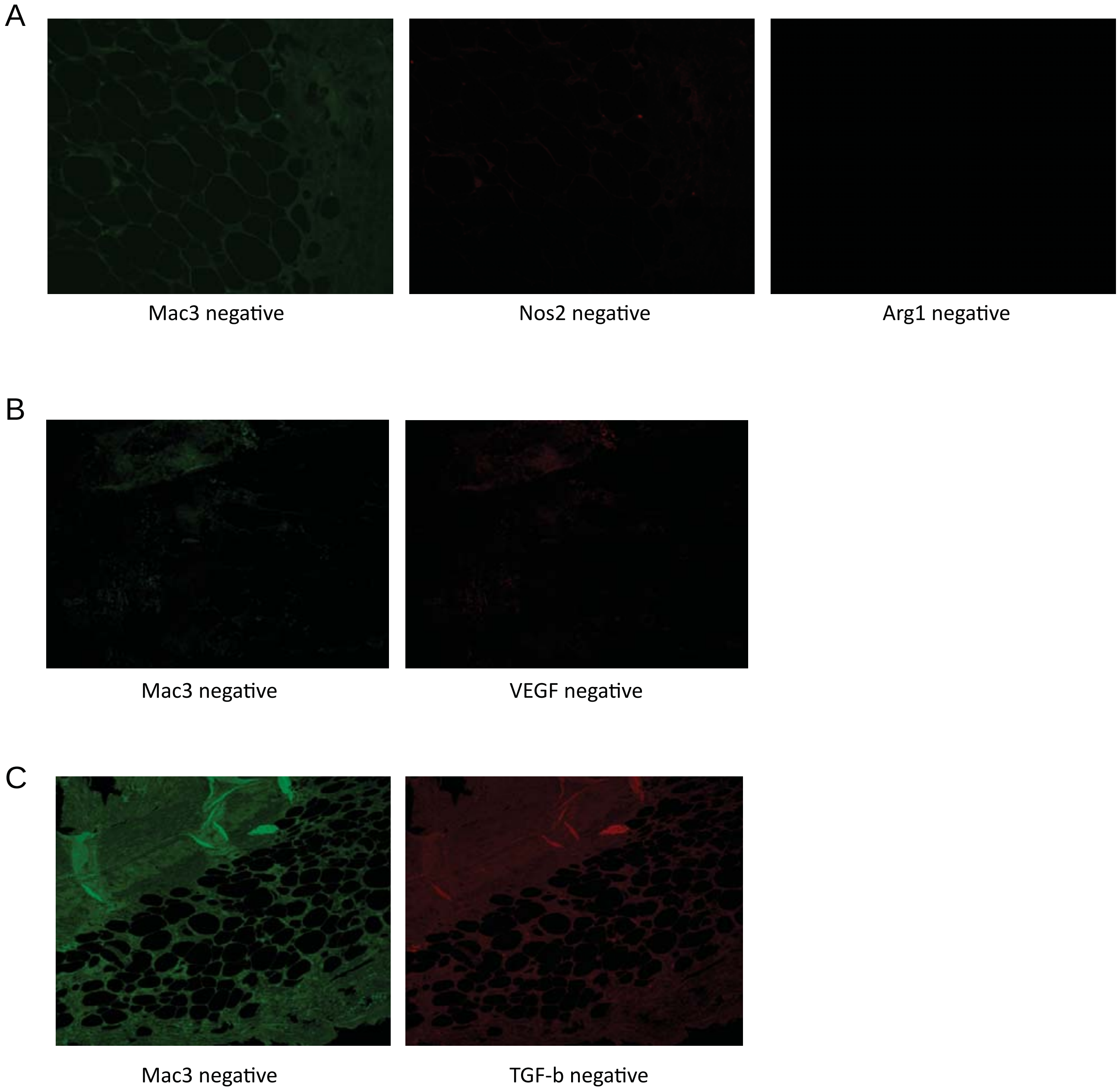

Supplementary Figure 3. Immunofluorescent controls. Negative control (secondary only) staining for Mac3, Nos2, and Arg1 in (A), Mac3 and VEGF in (B) , Mac3 and TGFβ in (C) from sections of day 7 wounds of db mice.
